# Playback‐Aided Surveys and Acoustic Monitoring in the Detection of the Endangered Forest Owlet *Athene blewitti*

**DOI:** 10.1002/ece3.70549

**Published:** 2024-11-14

**Authors:** Amrutha Rajan, Aditi Neema, Pranav G. Trivedi, Sejal Worah, Meera M. R., Shomita Mukherjee, V. V. Robin

**Affiliations:** ^1^ Indian Institute of Science Education and Research Tirupati Tirupati Andhra Pradesh India; ^2^ Salim Ali Centre for Ornithology (SACON) ‐ South India Centre of WII Coimbatore India; ^3^ Independent Consultant Ahmedabad India; ^4^ WWF‐India New Delhi India

**Keywords:** automated recording units, bioacoustics detection, conservation, endangered bird, landscape change, resurveys, Western Ghats

## Abstract

Monitoring rare and endangered species over the long term is challenging due to limited historical data and comparable methods. Climate and landscape changes can significantly impact species distributions, driving some to extinction. The Forest Owlet is an endangered bird considered extinct but rediscovered after 113 years in 1997. Since its rediscovery, followed by the description of its calls, there have been regular recent sightings of the species from newer locations, leading to its downlisting in the IUCN Red List from critically endangered to endangered. In the Dang region in Gujarat, India, there have been no historical records despite previous systematic ornithological studies over three decades, but have multiple sightings over the last few years. Although we now know a little more about the broad association of the species occurrence with habitat and climate variables, a major focus of this study is to estimate the reasons for the “appearance” of the Forest Owlet in Dangs. We revisited locations of past surveys to determine if the species is currently found in the study sites where it was previously unrecorded. We also examine whether new survey methods using playback of its call could enhance its detection. During resurveys, we located the Forest Owlet at new, previously unrecorded locations. Analyses of satellite imagery products revealed significant changes in the broader Dang landscape, including the loss of native forests, increased agriculture, and shifts in mean maximum temperature and rainfall. Our research suggests playback can enhance detection, although its effectiveness varies across landscapes. A detection strategy for long‐term monitoring was developed using different acoustic detectors. An effective detection distance of 300 m within the habitat was achieved using automated recording units (ARUs). Although the species responds to climate and habitat change, the cause of the increased reports of this endangered species remains undetermined. However, we found increased detections using newer survey techniques involving bioacoustics. We recommend using these techniques carefully for future baseline studies, which are urgently required.

## Graphical abstract

Long‐term monitoring of populations of rare, endangered species is often challenging due to the lack of baseline historical datasets and appropriate methods. The current study proposes a detection framework that includes the newer survey techniques with bioacoustics which showcase an increase in the detection of the endangered Forest Owlet from the past unrecorded sites.

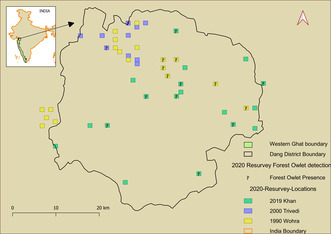

## 1 Introduction

Anthropogenic climate change and land‐use changes are known to have significantly affected biodiversity (Blake and Loiselle 2015; Mantyka‐Pringle et al. 2015; Skogen, Helland, and Kaltenborn 2018). We are limited in our ability to detect such changes in biodiversity due to the lack of appropriate comparative datasets or challenges with detecting cryptic species. Many cryptic species escape detection through traditional surveys for lack of appropriate search images (Lauriault and Wiersma 2019), and in modern times are enhanced through the use of camera traps (Thomas et al. 2020), and automated recording units (ARU) (Bobay, Taillie, and Moorman 2018; Frommolt and Tauchert 2014; Zwart et al. 2014) deployed over longer periods to increase the chances of detecting such species.

The best‐known examples of recent resurvey of avian diversity are the comparisons with the early 20th century Joseph Grinnell surveys indicating a loss of up to 43% of species (Iknayan and Beissinger 2018; Tingley et al. 2012; Tingley and Beissinger 2013). They also indicated that the Mojave bird community collapse was affected by climate change, chiefly through precipitation changes rather than temperature. A key element of any resurvey is the availability of systematic historical data that can be adapted to modern methods of biodiversity surveys. From the biodiversity hotspots of India, there are very few such datasets and even fewer resurveys like Sashikumar et al. (2014) and Subramanya (2019).

**FIGURE 1:**
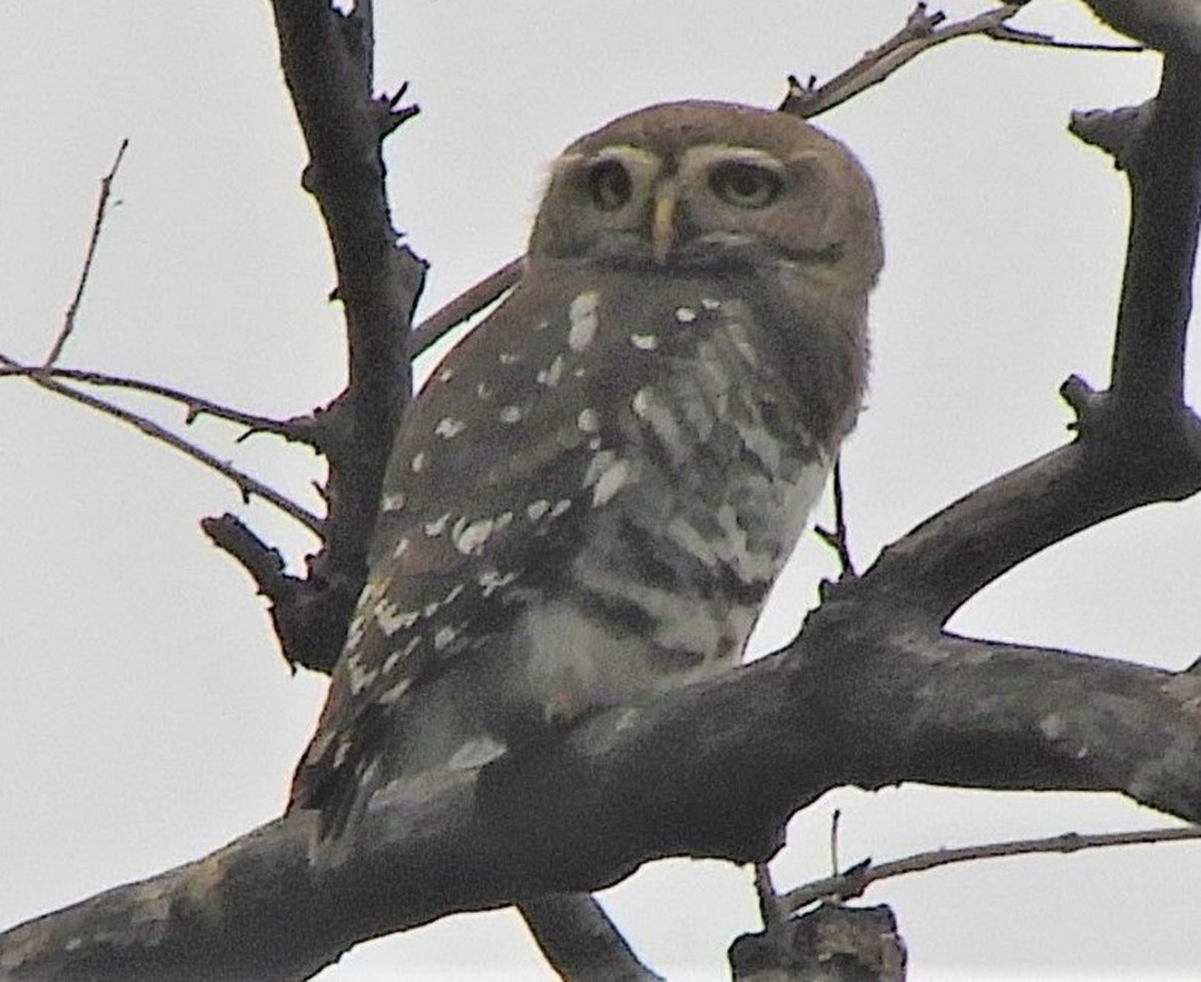
Image of the Forest Owlet captured from the Dang Study area (Image credit: Amrutha Rajan).

The Forest Owlet *Athene blewitti* is endemic to India, occurring only in small parts of central India and the northern Western Ghats (BirdLife International 2018). After 113 years, it was rediscovered in 1997 in north Maharashtra (King and Rasmussen 1998). Since its rediscovery, new records have emerged at various locations in Madhya Pradesh, Maharashtra, and Gujarat (Mehta et al. 2017). Until 2017, its population was assessed to be less than 250 individuals remaining in the wild and was listed as a critically endangered species (BirdLife International 2016). However, the population is now estimated to be up to a thousand individuals, and the species was downlisted as an endangered species in 2017 (BirdLife International 2018). The Forest Owlet appears gray brown in color with white patches in the lower and upper belly and white prominent eyebrows, and the lack of white spots in the head is the major distinguishing feature from the Spotted Owlet *Athene brama* (Figure 1).

Most significantly, the first recorded call and three types of vocalizations of the species were described recently (Ishtiaq and Rahmani 2004; Rasmussen and Ishtiaq 1999). The location records of this species have increased since the recordings of its vocalizations have become more accessible. Today, playback of its calls is used in various detection‐based studies (Khan et al. 2023; Mehta et al. 2008, 2017).

The Forest Owlet's habitat association and occurrence have been extensively studied across the entire range of its occurrence (Khan et al. 2023). Khan et al. (2023) laid grids over high probability niche (climate and habitat) clusters covering 2.5% of the global distribution to assess the habitat relationships of the Forest Owlet. In addition, Khan et al. (2023) discovered that the average occupancy of the Forest Owlet was higher in the Dangs region, which has small patches of teak plantations and disturbed forests. This presented a curious circumstance where an endangered species had a fairly high occupancy but was not recorded in previous systematic surveys 3 decades back. In this study we proposed to resurvey previously assessed locations and examine factors such as landscape and climate change in the broader region of interest, apart from supplementing acoustics‐based detection methods for the long‐term and targeted monitoring of the species.

The development of automated recording units (ARU) and their Sound recognition algorithms have enabled researchers to conduct long bioacoustic deployments. The clear advantage of using bioacoustic sensors is obtaining long‐deployment data with vocalizations of rare species that may be difficult to detect with infrequent human visits. However, one of the challenges that remain with using ARU‐generated bioacoustic data is the validation of the presence of a particular species and the assessment of uncertainty around the detection of that species. Investigators have shown that background noise, the presence of other sonically similar species can obfuscate the presence of a target species (Ralph et al. 1993). The detection distance of a species in a specific habitat can impact detection and needs to be ascertained for each study (Haupert, Sèbe, and Sueur 2023; Piercy et al. 2014). Previously, a study showed the Forest Owlet call can be heard at a distance of 1 km (Ishtiaq and Rahmani 2004); however, this species has a variety of vocalizations that have yet to be assessed systematically. ARUs have been used to detect several endangered species (Arvind et al. 2023), and this is proving to be an efficient tool for the rapid assessment of threatened taxa.

One of the areas where the Forest Owlet is being increasingly recorded is the Dang area in Gujarat (Khan et al. 2023), at the northern edge of the Western Ghats. The first records of Forest Owlet from Dang were in 2014 (Patel et al. 2015), and since then, they have been increasingly recorded from this region. Although Salim Ali's landscape‐wide bird surveys in 1954 (Ali 1954) covered the Dang, Forest Owlet was not detected here, and neither have ornithological studies in 1990 (Worah 1991) and 2000 (Trivedi 2006). It is unclear if these birds have recently moved into this landscape or if the recent targeted surveys with different methods have enhanced the detection of the species.

Although it may not be possible to answer this question comprehensively, we revisited locations of two previous datasets of surveys from 1990 (Worah 1991), 2000 (Trivedi 2006), and a recent occupancy survey in 2019 (Khan et al. 2023) and resurveyed these locations with traditional and acoustic‐aided methods.

In this study, we use a cascading set of questions to examine the reasons for the changes in Forest Owlet detections over the years at a location—Dang Forests in Gujarat, while exploring different detection methods for future surveys.

First, we ask (a) Do Forest Owlet currently occur at sites that were previously surveyed thrice in the past (1990 to 2019).

Next, we ask (b) If detections of the Forest Owlet at the resurvey locations are similar with playback (newer methods) and without (traditional survey methods).

We also ask (c) If the landscape or climate has changed in the period between the surveys in the broader Dang region.

Based on the role of acoustics in detections, we further assessed the efficacy of using bioacoustics‐based detection of the Forest Owlet by asking:

(d) Which acoustic analysis software performs better at detecting the calls and songs of the Forest Owlet with co‐occurring owls.

And (e) At what distance should automated recording units (ARU) be placed to record the species in its habitat?

## 2 Methods

**FIGURE 2:**
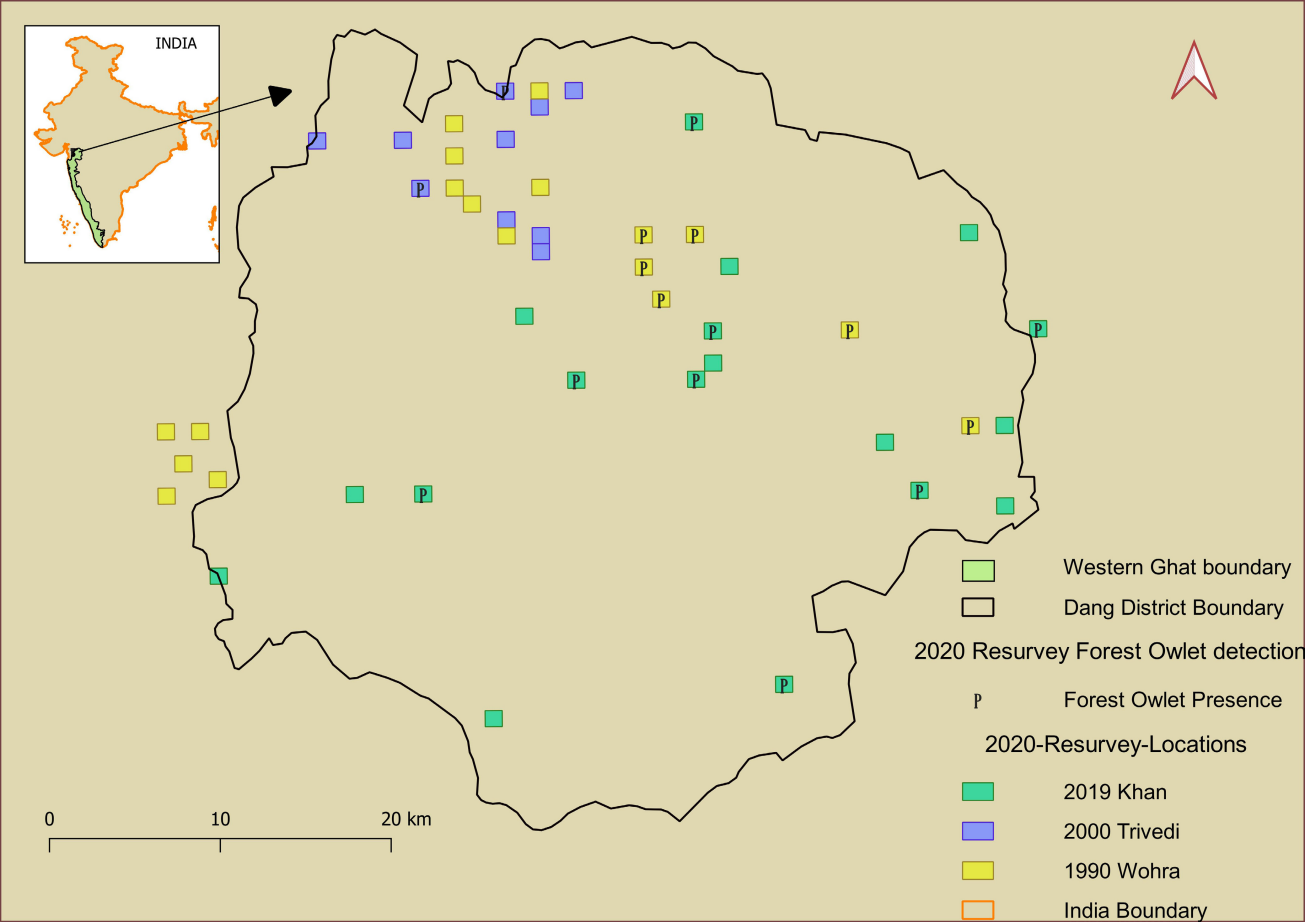
Resurveys for the endangered Forest Owlet in the Dang region result in detections at locations where studies 3 decades back did not.

This study was conducted in the northernmost part of the Western Ghats—Dang district of Gujarat (20°33′40″, 21°5′10″ North and 73°27′58″, 73°56′36″ East) (Figure 2), where a large portion (1035 sq. km) of the 1764 sq. km. is forested, and is spread across elevations ranging between 300 and 700 MSL.

- Do Forest Owlets currently occur at sites that were previously surveyed?

We collated three past datasets across which we based our resurveys. The earliest dataset was from 1988 to 1991 (Worah 1991), denoted hereafter as Worah 1990 by the year of the survey; and a decade later, in 2000 (Trivedi 2006), hereafter Trivedi 2000; and the latest, almost two decades later in 2018–2019 by (Khan et al. 2023)—hereafter Khan 2019. All study years are denoted by the year of surveys and not by the year of publication.

To assess any changes in climate and landscape, along with changes in the presence of the Forest Owlet, we also collated secondary datasets available for this region. Since climate and landscape change can have effects at a larger scale than the immediate location of occurrence, we included a buffer of 5 km around the Dang region—thus estimating the change in climate and landscape over 8245 sq. km. Given the limitation with the numbers of historical survey locations (Figure 2), we did not attempt to compare climate or landscape change at each survey site (grid—described below).

**Historical datasets assessed:**

- *Worah 1990* was a study of the entire bird community, without a specific search image for the Forest Owlet since it was not recorded from this region at that time. Worah used 5‐min counts of all birds that were seen or heard across 74 sq. km. of the landscape over 3 years (1988–1991).
- *Trivedi 2000* used 10 line transects (1 to 3.5 km length, open width) across ~160.84 sq. km to conduct bird counts through the year for 2 years (2001–2003) in the same landscape. During this survey too, Forest Owlets were not recorded from the landscape, and no particular effort was made to look for the species.
- *Khan 2019* is not a historical dataset, but these surveys were conducted between November 2018 to January 2019, specifically looking for Forest Owlet with active playbacks of the species. They surveyed 45 randomly selected grids across the Dang area, covering 2.5% of the total area. These surveys targeted detecting the Forest Owlet, and the team played back calls of Forest Owlet for 5 min (or until detection if that was earlier). They walked through the entire grid and conducted replicated (three or four) surveys over 2 days in each grid in the morning (6:00 h. to 10:00 h.) and evening (15:30 h to 19:00 h). Detailed methods are available in Khan et al. (2023). We included this dataset as a reference for a more extensive, focused study to compare our results with.

**Adapting previous surveys for the resurvey:**

Since the methods used in the past varied, we created a common spatial sampling framework. Worah 1990 did not have GPS locations of the specific points sampled but had detailed maps of sampled forest patches (Figure S1). We digitized these maps in consultation with Worah. Trivedi 2000 had spatial locations for each transect (Figure S2), but these were variable in length. Khan 2019 had 45 grids of 1 × 1 km that were sampled. We created 1 × 1 km grids across the landscape to encompass all three sampling strategies. This covered the seven broad locations of the Worah 1990 study, all 10 transects of Trivedi 2000, and overlapped with grids from Khan 2019 (including 18 grids where they detected Forest Owlets). Since the transects of Trivedi 2000 encompass multiple adjacent grids, we picked 18 random nonadjacent grids for the resurvey. Together they constituted a total of 46 grids of 1 km × 1 km that we selected to resurvey (Figure 2).

b Are detections of the Forest Owlets at the resurvey locations similar with playback and without?

### 2.1 Traditional Point Count Method—Without Playback

We attempted to recreate the sampling strategy used in Worah 1990 and Trivedi 2000 by visually searching and listening for the call of the Forest Owlet without any playback for 30 min within each grid. Two observers A.N. and A.R. worked together to cover the entire grid. Before starting this study, both observers were familiar with the species and its calls. This method was carried out before any playback was conducted in the region. Although this method recreates the spatial coverage of the sampled area, it only partially recreates the historic survey method since the resurvey focused on detecting a focal species with a search image. Such a specific search image may also affect the detection of species (Murton 1971; Suzuki 2018).

### 2.2 Call Playback Method

If the Forest Owlet was not detected using the traditional survey method at the same grid, playback of its calls and songs was carried out. The playback procedure followed was the same as Khan 2019, with the same set of calls (A.N. was part of both field studies). Briefly, playback was conducted at the center of each grid. If the centroid was inaccessible, a point close to the center within a 200 m buffer was selected. Playback was conducted for 5 minutes, followed by an active listening phase of 5 min and another final set of playback for five more minutes (a total duration of 15 min). At the end of the second playback, the survey was deemed complete if no detection was made earlier. The survey was discontinued for the grid in case the Forest Owlet was detected at any point during this survey period. All calls were played back using a portable Bluetooth speaker, Sony SRS‐XB10 connected to a digital recorder (Zoom H1) with a maximum volume of both the speaker and recorder set to match the natural call amplitude of the species (Darras et al. 2018).

All resurveys were carried out between January 2020 and March 2020.

c Has the landscape or climate changed in the period between the surveys?

**Detecting Landscape Change between the Survey Periods:**

We used two methods to detect the landscape change between the historical surveys and the current resurveys. First, we used the Global Forest Change v1.7 (2000–2019) dataset (Hansen et al. 2013). The forest cover loss for the Dang area (with a 5 km buffer as described earlier) was extracted in Google Earth Engine (Gorelick et al. 2017). The authors (Hansen et al. 2013) defined Forest Cover Loss as a change from a forest to a nonforest state (from 2000 to 2019), where the term “forest” refers to tree cover and not land use, and trees were defined as all vegetation taller than 5 m in height.

Furthermore, we used data from Roy et al. (2015) that mapped the Land‐Use and Land‐Cover Change in India at decadal intervals for 1985, 1995, and 2005 using the data extracted from multiple imageries and classified following the classification scheme of the International Geosphere‐Biosphere Programme (IGBP) (Loveland et al. 2000). We used the Lacos plugin in QGIS (Jung 2013) to calculate the change in land‐cover classes in the resurvey grids and across the Dang district, including the 5 km buffer zone described previously. Nine landscape classes occurred in the study area of Roy et al. (2015) (details in Appendix S1), and we assessed changes in each of them relevant to the project. We ran an unpaired two‐samples *t*‐test to test the significance of landcover change between 1985 and 2005 across grids in the broad Dang region (Figure 4).

Climate change was examined from 1980 to 2019 across the Dang region within the same 5 km. buffer described earlier. We used two types of data to assess these changes.

First, we obtained climate data from Mishra et al. (2019) for the period 01‐01‐1951 to 31‐12‐2019. The resolution of this data is at 0.25°, so we selected the nine grids (Figure S3) that overlap with the Dang study area (Figure 2). For each grid, we collated data on the mean monthly minimum temperature, mean monthly maximum temperature, and monthly precipitation. We ran ANOVA to check the significance of these climate variable changes over the years of 1988, 2000, and 2019 based on the year's closest to the original survey period (Figure 5).

Furthermore, we also assessed change in climate using 19 bioclimatic variables from monthly climate data from Mishra et al. (2019) and two other sources for comparison: (a) Worldclim 2.5 min resolution and an average for the years 1951–2000 (Worldclim Climate Data Version 1.3 2004) and (b) Chelsea remotely sensed bioclimate data of average years 1981 to 2010 at a resolution of 30 arcsec (926 m) (Karger et al. 2021) (Figure S7). We used the biovars function from the *dismo* package (Hijmans et al. 2017) in R. We plotted the spatial changes of selected bioclimatic variables derived from Chelsea's average bioclimate data of 1981–2010 (Figure S6) across the Dangs region. These bioclimatic variables—BIO10 (Mean temperature of the warmest quarter), BIO11 (mean temperature of the coldest quarter), and BIO15 (precipitation seasonality [coefficient of variation]) were considered important for Forest Owlet presence, based on the niche models by Koparde et al. (2019) (Figure S6).

**Establishment of an acoustic detection framework:**

d Which acoustic analysis software performs better at detecting the calls and songs of the Forest Owlet with co‐occurring owls?

### 2.3 Data Collection

All acoustic data were collected during the Forest Owlet resurvey study period (January 2020 to March 2020), and all data was recorded between morning 06:00 to 10:00 h and evening 14:00 to 18.30 h. We collected data with two types of recorders. We used handheld recorders (Zoom H6, F1) to collect several clear target recordings of Forest Owlet vocalizations for training signal recognition software. We also used an automated recording unit (ARU) (Song Meter SM2; Wildlife Acoustics Inc., USA) as a passive recorder, collecting long‐term data for testing the Forest Owlet detection in both signal recognition software (described further below).

### 2.4 Vocalizations of Forest Owlet

**FIGURE 3:**
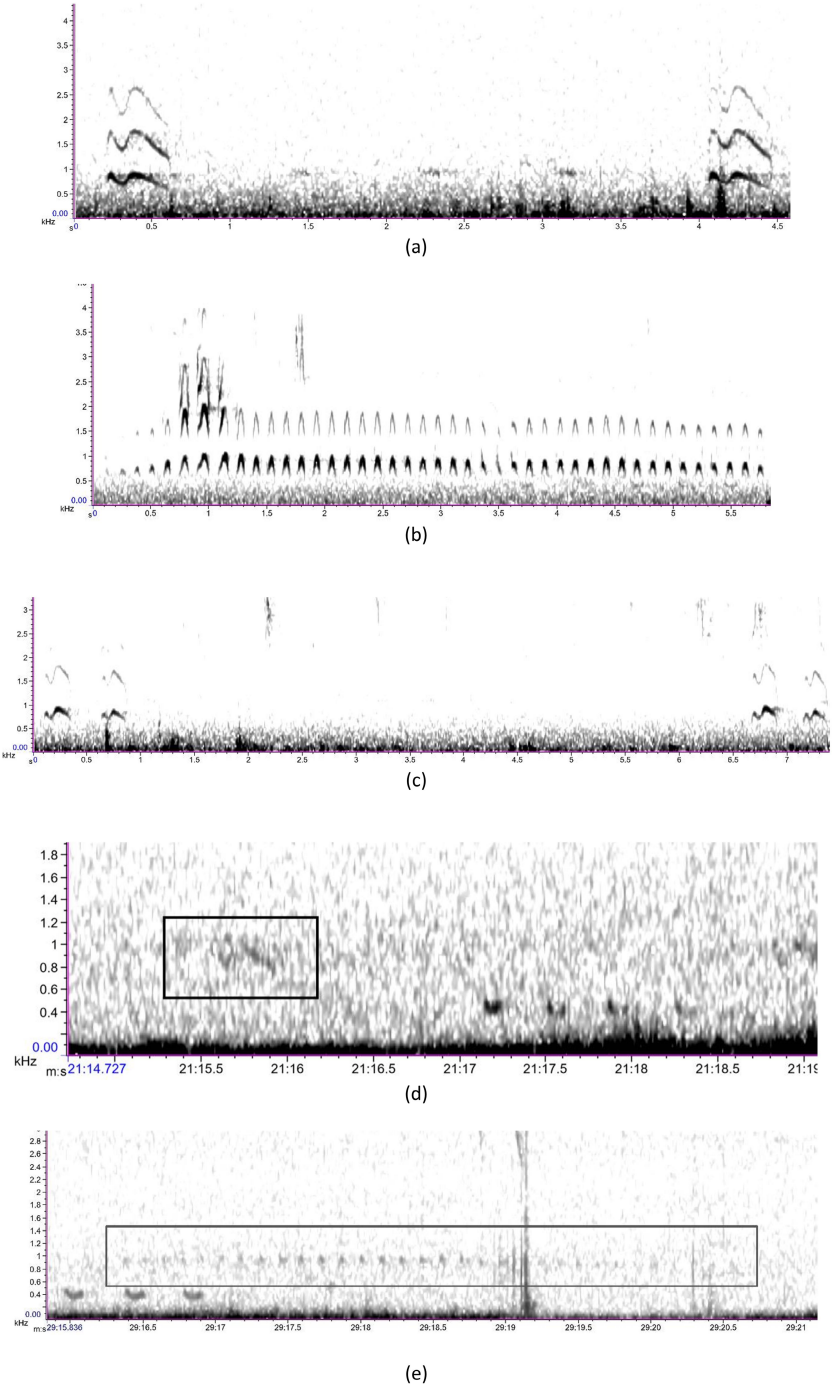
Spectrogram of Forest Owlet vocalizations—(a) kuhuk calls, (b) kwak kawak songs, (c) a variation of kuhuk call, and Spectrogram view of Forest Owlet call (d) and song (e) detection in SM2 recorder at a distance of 300 m from the vocalizing bird on the field (represented inside the box).

We used two vocalizations of Forest Owlet to test the detector's efficiency. Additionally, we found a variant of one of the vocalizations while collecting high‐quality recordings from handheld recorders in the field. We denote the two main vocalizations as call and song. Call is disyllabic and has a frequency of around 500 to 1100 Hz, (read as “kuhuk”). The song is monosyllabic with a frequency of around 400 to 1400 Hz and longer than the call (read as “kwaak…kakak, kwaak…kakak”) (Figure 3).

### 2.5 Forest Owlet Detection Using Signal Detectors

Automated acoustic signal recognition software functions by processing a training template of the target species' vocalization following the recognition and detection based on the parameters chosen. We selected two signal‐detecting software, Raven Pro 2.0 (Bioacoustics Research Program, K. Lisa Yang Center for Conservation Bioacoustics 2018) and Kaleidoscope (Kaleidoscope Pro Analysis Software 5.6.6 2024) to create a detector to test the accuracy in detecting the Forest Owlet vocalizations from the field recordings. The recordings collected by the passive recorders (Song Meter SM2; Wildlife Acoustics Inc., USA) during the detection distance estimations were used as a test dataset to run the signal‐detecting software for the Forest Owlet detection. A combination of handheld recorder, and ARU data of Forest Owlet vocalizations collected were used as training dataset.

- Raven Pro 2.0—Template Detector

Raven Pro 2.0, the acoustic analysis software, requires a good‐quality spectrogram template; we obtained these from recordings with handheld recorders (Zomm F1, H6). The template detector function of Raven is used to run the test detection of Forest Owlet vocalization. Effective signal‐detecting parameters in Raven were chosen based on the F‐Beta values (Arvind et al. 2023; Nolan et al. 2023). F‐Beta is the harmonic mean between the precision and recall values of the detector. Here, we selected one as the Beta value to measure the F‐Beta and referred to it as F‐1 Score, which indicates that we gave equal importance to the precision and recall values of the detector performance (Nolan et al. 2023). Based on the F‐1 score values, we selected the threshold and frequency values that were better to obtain higher detection rates of Forest Owlet vocalization. The F‐1 score is calculated by,

$$F-1\;\text{score}=\left(1+\beta^{2}\right)\;{\text{Precision}}^{*}\text{Recall}/\beta^{2*}\;\text{Precision}+\text{Recall}$$

Based on the results of the F‐1 Score analysis, we selected specific spectrogram parameters in Raven 2.0 for running the template detector algorithm, such as window size at 1024, the optimum threshold at 0.65, a frequency of 80 Hz for the Forest Owlet call, and an optimum threshold at 0.45 and a frequency of 100 Hz.

b Kaleidoscope—Advanced Classifiers

The recordings were analyzed by Kaleidoscope Software (version 5.6.6) from Wildlife Acoustics. Kaleidoscope is a signal detection recognizer that builds a classification algorithm by running individual call syllables through Hidden Markov Models (HMM) that maximize the probability of detecting the entire call structure. Kaleidoscope uses K‐means clustering of Fisher Scores from a 12‐state HMM to cluster all the signals detected into different classes, as opposed to only identifying the signals that match the algorithm above a user‐set score threshold (Knight et al. 2017). Advanced cluster analysis was used in the Kaleidoscope detector to reduce the false positive rates since the focus was to detect specific individual species by grouping similar vocalizations in a cluster. This includes a two‐step training process and the final testing with the original long‐term field recording. The initial two steps involve training the detector by providing good‐quality recordings of the Forest Owlet vocalizations from handheld recorders (Zoom H6, F1) to select the appropriate settings (Table 1) followed by a trial run for the better test detection.

**TABLE 1:** Features used for the detection of Forest Owlet vocalizations in the Kaleidoscope detector.

Features used	Call	Song
Frequency range	500–3500 Hz	300–2000 Hz
Length of detection	0.1–0.5 s	0.1–7.5 s
Max intersyllable gap	0.35 s	0.35 s
Max distance	0.5	1.0
FFT	512	512
Max states	12	12
Max distance to cluster center	0.5	0.5
Max clusters	30	50

The frequency range was selected to encompass the target calls, and the length of detection and intersyllable and max distance were based on the two types of calls. The number of clusters was set to be larger for kwaak since these are longer songs.

In summary, the process involved in the detection of Forest Owlets using signal detectors:

- Training the algorithm (handheld recording data for both detectors)

1. Raven—5 bouts of each vocalization.
2. Kaleidoscope—250 files ranging from 5 s to 15 min.

The difference in the training data is due to the differential requirements of the template detector‐based Raven (Bioacoustics Research Program, K. Lisa Yang Center for Conservation Bioacoustics 2018) that requires high‐quality targeted recordings versus the clustering algorithm‐based Kaleidoscope (Kaleidoscope Pro Analysis Software 5.6.6 2024) that requires data as close to the test data. b. Trial—A trial run of both detectors against 1 h of ARU data to choose the appropriate settings for better detection. c. Test—We ran both detectors against 7‐h ARU data with chosen parameters based on the trial findings.

### 2.6 Detector Performance Analysis

The efficiency of both detectors was tested with a common test dataset of a 7‐h recording collected during our field survey. The efficiency of detectors was measured in terms of F‐1 score values.

e What is the detection distance for automated recording units (ARU) to record the Forest Owlet in its habitat?

We tested the detection distance of the Forest Owlet in the wild. First, we elicited an acoustic response from the Forest Owlet by doing a playback of two vocalizations in the center of the selected grid. We identified the perch location based on the vocalizing individuals prior to deploying the ARUs in16 positive Forest Owlet grids. We placed two to three recorders at a time from the Forest Owlet vocalizing perch to eventually cover several distances, 100 m, 200 m, 300 m, 400 m, 500 m, 600 m, and 1000 m. We changed the ARUs accordingly if the bird shifted the perch location, and new vocalizing perch location if it was still vocalizing. Most often, Forest Owlet stayed at a perch while vocalizing. Recording units were programmed to record continuously from deployment with a sample rate at 44100 Hz in Mono using the WAV format with a bit depth of 16 and a gain level between −12 dB to 22 dB. The Forest Owlet detections from the recordings of the detection distance experiment were confirmed by the manual visual inspection of spectrograms and annotating each vocalizations in the Raven Pro 1.6 (Figure 3d,e).

## 3 Results

In this study, we resurveyed 46 grids of 1 km × 1 km that were previously surveyed at different times in the last 2 decades. We examined the presence of the Forest Owlet with both the traditional and the more recent playback‐based techniques.

### 3.1 Forest Owlets Detection at Sites That Were Previously Surveyed, Differences With Playback and Without

We detected the Forest Owlet from the areas represented in all three studies, including areas where they were previously not recorded. Additionally, at some of the historic nondetection sites, we could detect Forest Owlet using the traditional visual survey methods (without playback), but the number of detections with playback was considerably higher. We detected the Forest Owlet in 16 grids of the 46 resurveyed. The resurvey of the 18 grids from Khan 2019 with Forest Owlet detections resulted in eight grids with detections in this study (Table 2 and Figure 2). Eight of the 28 grids with no previous Forest Owlet detections (traditional surveys) had Forest Owlet detections during this study.

**TABLE 2:** Details of resurveys, effort, and detections of the Forest Owlet in the Dang region.

Surveys by other investigators (details of original surveys in col no. 1)			Resurveys (2020)—this study		
Team and year of survey	No. of grids originally surveyed (A)	Grids (from A) in which Forest Owlet was recorded	No. of grids (from A) resurveyed (B)	No. of grids Forest Owlet detected during resurvey (from B) without playback.	No. of grids Forest Owlet detected during resurvey (from B) with playback
Khan 2019	45	18	18	2	6
Trivedi 2000	11	0	10	1	1
Worah 1990	74	0	18	0	6

### 3.2 Landscape Change Analysis

**FIGURE 4:**
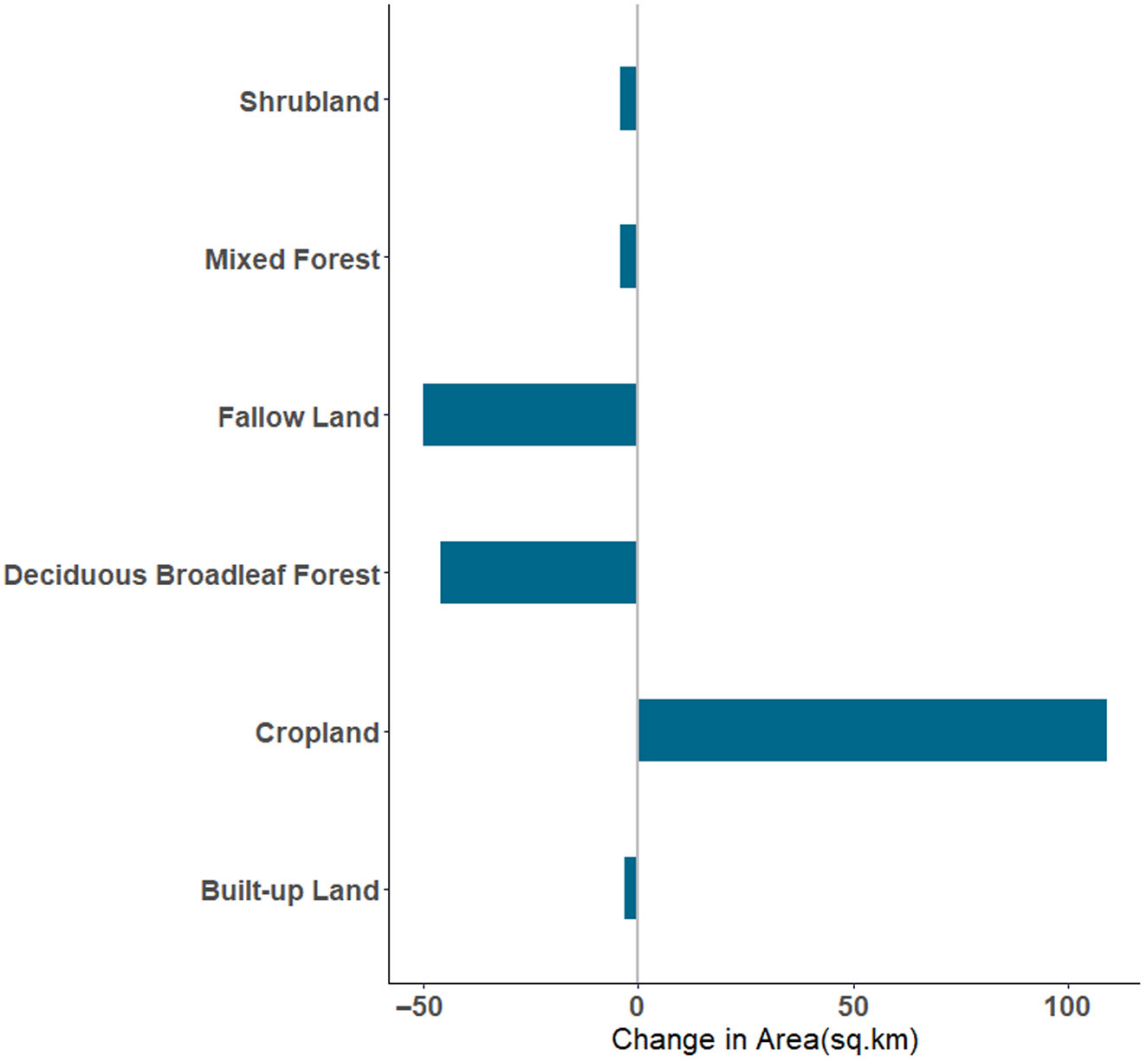
Change in the area (sq. km) of land‐use types from 1985 to 2005 across the Dang region based on data from Roy et al. (2015) with a 5 km buffer indicating landscape change at a larger landscape scale are significantly different (unpaired two‐samples *t*‐test, *p* = 0.0007695) (classes described in detail in Appendix S1).

- Forest cover change based on Hansen et al. (2013) data: Our assessments of forest cover change over the last 2 decades across the entire study area of Dang showed an overall minimal decrease of 0.4446 sq. km (Figure S5), when assessed with global data products (Hansen et al. 2013).
- Change in the land‐cover area based on Roy et al. (2015) data:

In the Dang study area, we found a significant change (*p* = 0.0007695) in the land use, with a 109 sq. km increase in cropland (20% of category) and a decrease in about 46 sq. km of Deciduous Broadleaf Forests (63% of category) and 50 sq.km Fallow land (0.7% of category) occurred between 1985 and 2005 (Figure 4). Shrubland and Mixed Forest also show a minor decrease in area, and Wasteland shows a minor increase (Figure 4). Although Barren Land and Built‐up land were also identified in the study area, no change was detected in this area during this period (Figure 4). We found a significance difference between the five landcover class (Figure 4) across the broad Dang region (with a 5 km buffer Figure S4) between 1985 and 2005 (*p* = 0.0007695).

### 3.3 Climate Change Analysis

**FIGURE 5:**
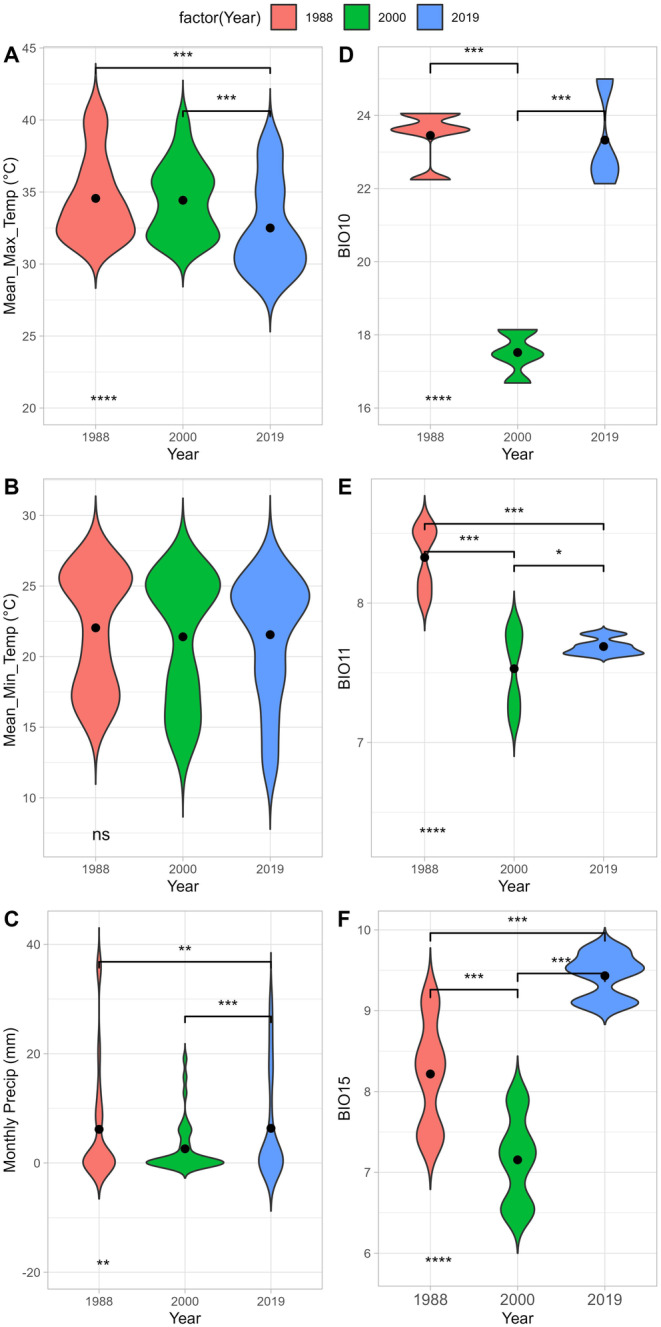
(A) Annual mean maximum temperature (°C), (B) Annual mean minimum temperature (°C), (C) Monthly mean Precipitation in mm, (D) BIO10, mean temperature of the warmest quarter, (E) BIO11, mean temperature of the coldest quarter, (F) BIO15, precipitation seasonality (coefficient of variation) across Dang (5 km buffer—9 climate points). * Indicates significance level (*p ≤ 0.05, **p ≤ 0.01, ***p ≤ 0.001, ****p ≤ 0.0001) (For the last two), black dot indicates the mean values.

Based on the datasets we examined, there were differences in climate across the survey years. We did not recover any major differences in annual mean minimum temperature over the survey years (Figure 5B), but there was a significant difference in the annual maximum temperature between 1988 and 2019, and 2000 to 2019 (*p* = 3.7e−08; Figure 5A). We also found a significant change in monthly precipitation between 2000 and 2019 (*p* = 0.001; Figure 5C). Precipitation seasonality (BIO 15) had significantly increased in 2019 compared with the previous survey years (*p* < 2.2e−16; Figure 5F). The mean temperature of the coldest quarter (BIO11) was significantly higher in 1988 compared with 2000 and 2019 (*p* < 2.2e−16; Figure 5E). The mean temperature of the warmest quarter (BIO10) is significantly lower in the year 2000 compared with 1988 to 2019 (*p* < 2.2e−16; Figure 5D).

### 3.4 Establishment of an Acoustic Detection Framework

#### 3.4.1 Detector Performance Analysis

**FIGURE 6:**
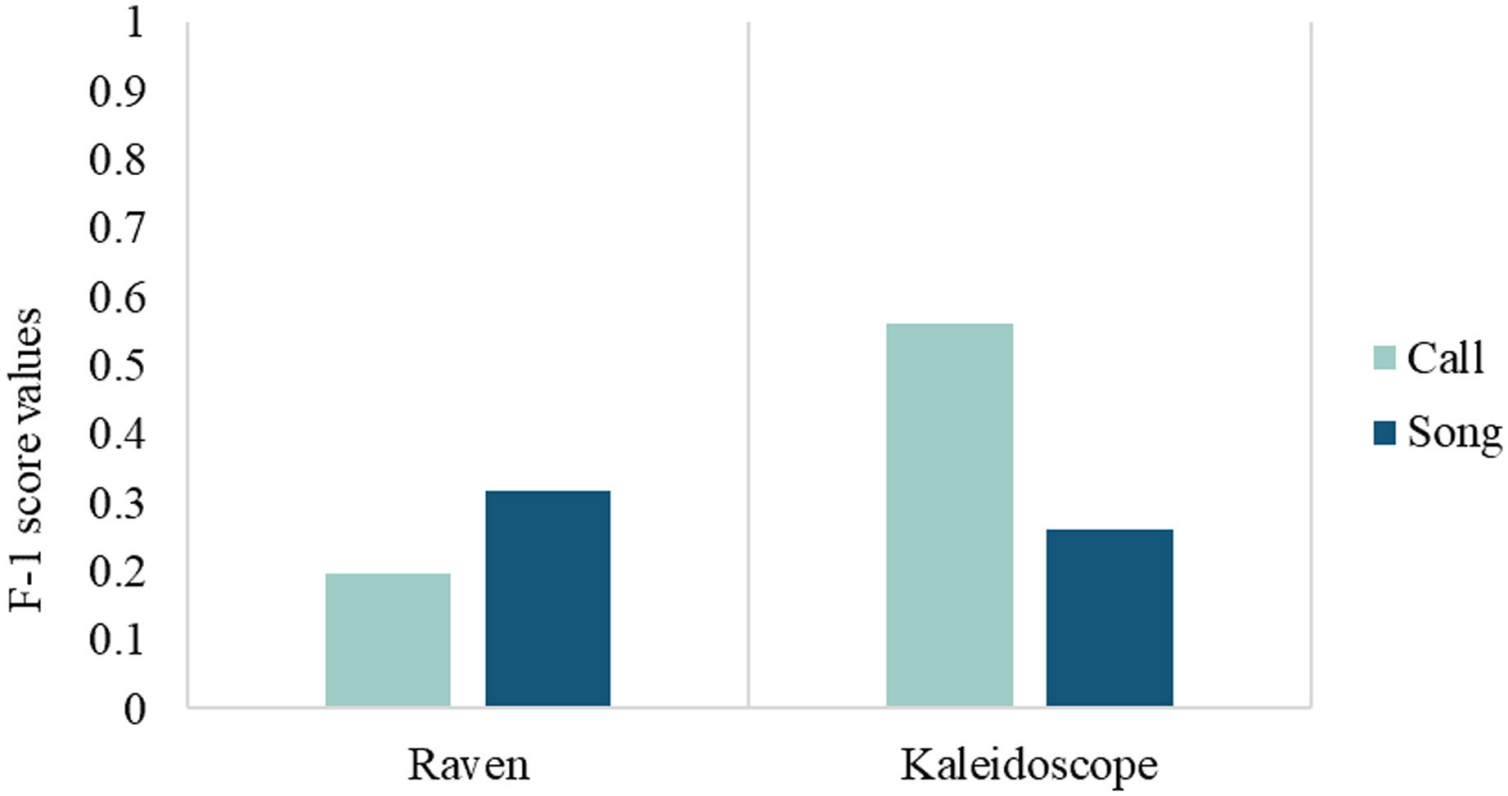
F‐1 score of Raven and Kaleidoscope in the detection of Forest Owlet vocalizations.

A total of 124 calls and 83 songs of the Forest Owlet vocalization were detected from the 7 h of field recording using automated recorders. We found that the Kaleidoscope detector performed better at detecting the shorter Forest Owlet call with a higher precision of 0.692, recall of 0.473, and F‐beta score of 0.562 (Figure 6). However, the Forest Owlet song was detected better in the Raven detector with a relatively higher precision of 0.465, recall of 0.24, and an F‐beta score of 0.31 (Figure 6).

#### 3.4.2 Detection Distance Analysis

We found that the Forest Owlet responded with its vocalization for a duration ranging from 1 min to 5 min following our call playback. By placing SM2 recorders from 100 to 1000 m at various intervals, we could detect the clear Forest Owlet acoustic vocalizations at a distance of up to 300 m from the real bird acoustic activity (Figure 3d,e).

## 4 Discussion

In this study, to assess the presence of the Forest Owlet, we resurveyed 46 sites (1 × 1 km grids) previously surveyed by multiple researchers. We detected the Forest Owlet in areas where previous studies had not reported them.

We consider three possibilities with varying survey methods—search image, playback, and temporal replicates and two possible biological reasons—landscape change and climate change for this outcome.

### 4.1 Role of a Search Image

Numerous studies have indicated that studies that are not actively searching for a target species, such as in surveys of broad communities, often miss cryptic species (Dawkins 1971; Lauriault and Wiersma 2019). In India, broad studies of Western Ghat birds surmised that the skulking rainforest bird, *Sholicola* was rare (Daniels et al. 1991; Kannan 1998) while targeted surveys found it to be common at high elevations (Robin and Sukumar 2002). Here, two datasets (Worah 1991) and (Trivedi 2006) were broad bird community studies from the landscape and were conducted with no search image of this target species at a time when the Forest Owlet was not known to occur in the landscape. Our detection of the Forest Owlet from the exact locations does throw up the possibility that the bird was present but not detected.

### 4.2 Role of Playback

Several bird detection studies have documented that the use of playback (of calls or songs) increased the detection probability relative to the passive surveys (Boscolo, Metzger, and Vielliard 2006; Celis‐Murillo, Deppe, and Allen 2009; Haug and Didiuk 1993). The detection of owls is more effective with call playbacks (Conway and Simon 2003; Haug and Didiuk 1993). The male burrowing owls *Speotyto cunicularia* were very responsive and immediately responded to the playback by singing back the primary song, bobbing, and flying toward the source (Haug and Didiuk 1993). Although we find increased detection and responses to playback, the behavioral ecology of this response is not very clear. The first information on the Forest Owlet vocalizations and behavior was provided by Rasmussen and Ishtiaq 1999, and only more recent surveys (Ishtiaq and Rahmani 2004; Khan et al. 2023; Mehta et al. 2008), were able to use this method. We note that most numbers of reports of the Forest Owlet have increased since the publication of its vocalizations. We note that our study was not designed in a neutral manner to test the effectiveness of playbacks since our goal was to compare previous methods with the current ones, but our data does suggest that playbacks may enhance detection.

### 4.3 Significance of Temporal Replicates

We find that even with playback, there is variation in the detection of this rare species. Khan et al. (2023) note that the detection probability of the species is ~70% in Dang with multiple temporal replicates. Of the 18 grids where they detected Forest Owlet in Dang, 13 (72%) grids had positive detections in all four of their survey replicates. However, just a year later, we did not detect them in all of these grids. Significantly, our study did not include temporal replicates, and some nondetection may be associated with detection probability. Most importantly, although there is stochasticity in the detection (or presence) of this relatively rare bird, even when the first temporal replicate of Khan et al. (2023) is considered, 18 of 45 grids were positive, which is similar to our detections (8 of 18 grids). This pattern holds even if we consider the identity of the grid. Within the design constraints of the current study, we cannot ascertain if this stochasticity is due to the probability of detection or a biological process such as local movement. This is where we suggest acoustic monitoring, described further below, comes in.

### 4.4 Relationship With Climate and Landscape Change Data

Numerous studies across the globe have found species occurrences responding to both climate and landscape changes. Resurveys of Iknayan and Beissinger (2018) in a desert landscape indicate that climate change may be related to the change in bird communities over the decades. However, in some cases, species like understory insectivorous birds are more vulnerable to land‐use changes (Sreekar et al. 2015). Our study finds some changes over the decades with the raw climate data and also with the derived/combined climate data. Some of these climatic variables were shown to be relevant for broader Forest Owlet distribution based on a model created by Koparde et al. (2019). However, both outputs need to be considered with the caveat of the spatial resolution and scale of the original data. Original climate data of Mishra et al. (2019), Worldclim and Chelsea are at very large scales (details in the methods section) and may not capture changes that occur at the scale of our study area. There are few ground sensors in this region to examine such patterns for this period and this remains a major caveat. Our study did not find any change in landscape to forest cover as measured with global products (Hansen et al. 2013). However, we do find changes based on a regional data product (Roy et al. 2015), and the changes are consistent with an increase in agriculture and a reduction in deciduous forests and fallow lands (indicative of open habitats). We note that field observations by Worah 1990 and Trivedi 2000 suggest that agriculture has increased, and the teak plantations have matured and become larger. Such details are not reflected in the current dataset and may well impact local site preferences. The type of agricultural practice—retaining old‐growth trees, may also have facilitated the presence of the Forest Owlet. Another caveat is the lack of microclimate data from each of these grids, and our assessment remains at the broader Dang region. We also cannot rule out associated biological processes like competition with co‐occurring owls—Jungle Owlet and Spotted Owlet, interacting with the habitat and climate change. Much of these remain to be examined.

From our assessments of the various detectors, the longer vocalizations of the Forest Owlet—the songs are better detected with RAVEN, while the shorter calls are detected with Kaleidoscope. This is consistent with the findings that each species may vary in the detection based on specific signatures (Scott Brandes 2008). In this case, the two types of vocalizations of a species are detected differently (Romero‐Mujalli et al. 2021). Our assessment of the use of bioacoustics as a survey method included an assessment of the detection distance of the bird's call in its habitat. We found that for calls and songs that are clear enough to be detected with RAVEN or Kaleidoscope, the recorders must be placed 300–400 m from the vocalizing Forest Owlet. We note that this distance differs from the human detection distance of up to ~1 km (Ishtiaq and Rahmani 2004), as the current detection distance is specifically for clear spectrograms that can be detected with these algorithms. This distance estimate is best suited for the deciduous forests of Dang; in more open landscapes, the distance may be larger. Based on the extensive surveys of Khan et al. (2023), we are unsure that the species may be found in denser forests. Hence, this distance (300 m radius) could be used as a conservative grid size for placing ARUs for long‐term acoustic monitoring.

### 4.5 Recommended Long‐Term Monitoring Techniques

- Considering the stochasticity/variability in the detection of the Forest Owlet, we recommend using temporal replicates to detect the species.
- The use of playback has increased very rapidly in recent efforts in bird detection surveys, and our study suggests that playback is useful for the detection of the Forest Owlet. However, several authors (Harris and Haskell 2013; Watson, Znidersic, and Craig 2019), indicate that excessive playback can cause abandonment of territories, higher levels of anxiety (Budka et al. 2019) and may impact other crucial behavioral activities (Harris and Haskell 2013). In other cases, some birds may not respond similarly to playback (de Lima and Roper 2009), and some may stop responding to excessive playback (Jepson 2011). Although our study did not explicitly test the impacts of playback, we do not recommend durations longer than 5 min at an amplitude louder than the natural calls and caution the use of playbacks without an appropriate study design.
- We recommend long‐term monitoring of the Forest Owlet using Automated Recording Units. These can also be deployed at likely Forest Owlet sites to understand the species' range better. We recommend a gird size of 600 m (based on a 300 m detection distance from this study) in deciduous forests.

In summary, we find Forest Owlet in locations where they were not previously detected, and we find a positive impact of using playback for surveys. We, however, do not attempt to make any causal links between the decadal detections of the endangered Forest Owlet and climate or landscape change. Although we note the changes indicated in climate and landscape over this duration, we suggest detailed monitoring of climate and landscape in these and surrounding areas for future monitoring. Based on the current study, we suggest survey protocols for the long‐term monitoring of the Forest Owlet.

## Author Contributions

**Amrutha Rajan:** data curation (equal), formal analysis (lead), investigation (equal), visualization (lead), writing – original draft (supporting), writing – review and editing (lead). **Aditi Neema:** data curation (equal), investigation (equal), writing – original draft (supporting), writing – review and editing (supporting). **Pranav G. Trivedi:** supervision (supporting), writing – review and editing (supporting). **Sejal Worah:** supervision (supporting), writing – review and editing (supporting). **Meera M. R.:** formal analysis (supporting), visualization (supporting), writing – original draft (supporting). **Shomita Mukherjee:** conceptualization (supporting), funding acquisition (equal), project administration (equal), resources (supporting), supervision (lead), writing – review and editing (supporting). **V. V. Robin:** conceptualization (lead), funding acquisition (equal), project administration (equal), resources (lead), supervision (equal), writing – original draft (lead), writing – review and editing (equal).

## Conflicts of Interest

The authors declare no conflicts of interest.

## Supporting information


Appendix S1.

## Data Availability

The data supporting the findings of this study are available within the article and/or its Appendix S1.
