# Supplementary material for: Playback‐Aided Surveys and Acoustic Monitoring in the Detection of the Endangered Forest Owlet Athene blewitti
Source: Ecol Evol. 2024 Nov 14;14(11):e70549. doi: 10.1002/ece3.70549 (PMC11563693; doi:10.1002/ece3.70549)
Supplement: Supplementary file 1 — Appendix S1. [file ECE3-14-e70549-s001.docx]

SUPPLEMENTARY MATERIALS

List of supplementary materials

1. Supplementary methods - land-cover classes from Roy et al. [(2015)](https://paperpile.com/c/VDaotW/2ET2k/?noauthor=1)*,*
2. Figure S1: Sampled areas of Worah 1990 study area across Dang
3. Figure S2: Locations of transects by Trivedi 2000 across Dang, including Purna Wildlife Sanctuary, North of Dang
4. Figure S3: Locations/centroids of 0.25-degree grids for which historic climate data was extracted for comparison with present data
5. Change in Climate
   - 1. Figure S4a: Annual mean minimum temperature across Dang over three time periods closest to the survey years shows no significant change
     2. Figure S4b: Annual mean maximum temperature across Dang over three time periods closest to the survey years shows a significant change in 2019
     3. Figure S4c: Mean annual precipitation across Dang over three time periods closest to the survey years shows no significant change
6. Figure S5: Forest loss across Dang from 2000 to 2019 appears minimal when assessed with global data products (Source: Hansen/UMD/Google/USGS/NASA)
7. Figure S6: Selected Chelsea Worldclim bioclimatic variables at locations with Forest Owlet detections across Dang region.
8. Figure S7: Chelsea (average of years 1981 to 2010) and Worldclim (average of years 1951 to 2000) selected the bio-climatic variable across 9 grids of climate locations from IIT-Guwahati climate data [(Mishra et al., 2019)](https://paperpile.com/c/VDaotW/UqNEz)

Supplementary methods:

   The land-cover classes identified in the study area by Roy *et al.* [(2015)](https://paperpile.com/c/VDaotW/2ET2k/?noauthor=1) are:

1. **Deciduous Broadleaf Forest** - Woody vegetation with a percent cover >60% and height exceeding 2 m. Consists of broadleaf tree communities with an annual cycle of leaf-on and leaf-off periods.
2. **Cropland** - Temporarily cropped area followed by harvest and a bare soil period (e.g. single and multiple cropping systems). Note that perennial woody crops will be classified as either forest or shrubland, whichever is appropriate. Includes orchards. Different types of croplands based on seasons (e.g. kharif, rabi, zaid) were not subclassified.
3. **Builtup Land** - Land covered by buildings and other man-made structures
4. **Mixed Forest** - Trees with a percent cover >60% and height exceeding 2 m. Consists of tree communities with interspersed mixtures or mosaics of the other four forest types. None of the forest types exceeds 60% of the landscape.
5. **Shrubland** - Land with woody vegetation less than 2 m in height and with greater than 10% shrub canopy cover. The shrub foliage can be either evergreen or deciduous
6. **Barren Land** - Exposed soil, sand, rocks, or snow and never have more than 10% vegetated cover during any time of the year
7. **Fallow Land** - Land taken up for cultivation temporarily allowed to remain uncultivated for one or more seasons.
8. **Waste Land** - Sparsely vegetated land with signs of erosion and land deformation that could be attributed to lack of appropriate water and soil management, or natural causes. These are land identified as currently underutilized and could be reclaimed for productive use with reasonable effort. Degraded forest (<10% tree cover) with signs of erosion is classified under wasteland
9. **Water Bodies** - Areas with surface water, either impounded in the form of ponds, lakes, and reservoirs or flowing as streams, rivers, etc. They can be either fresh or salt-water bodies.


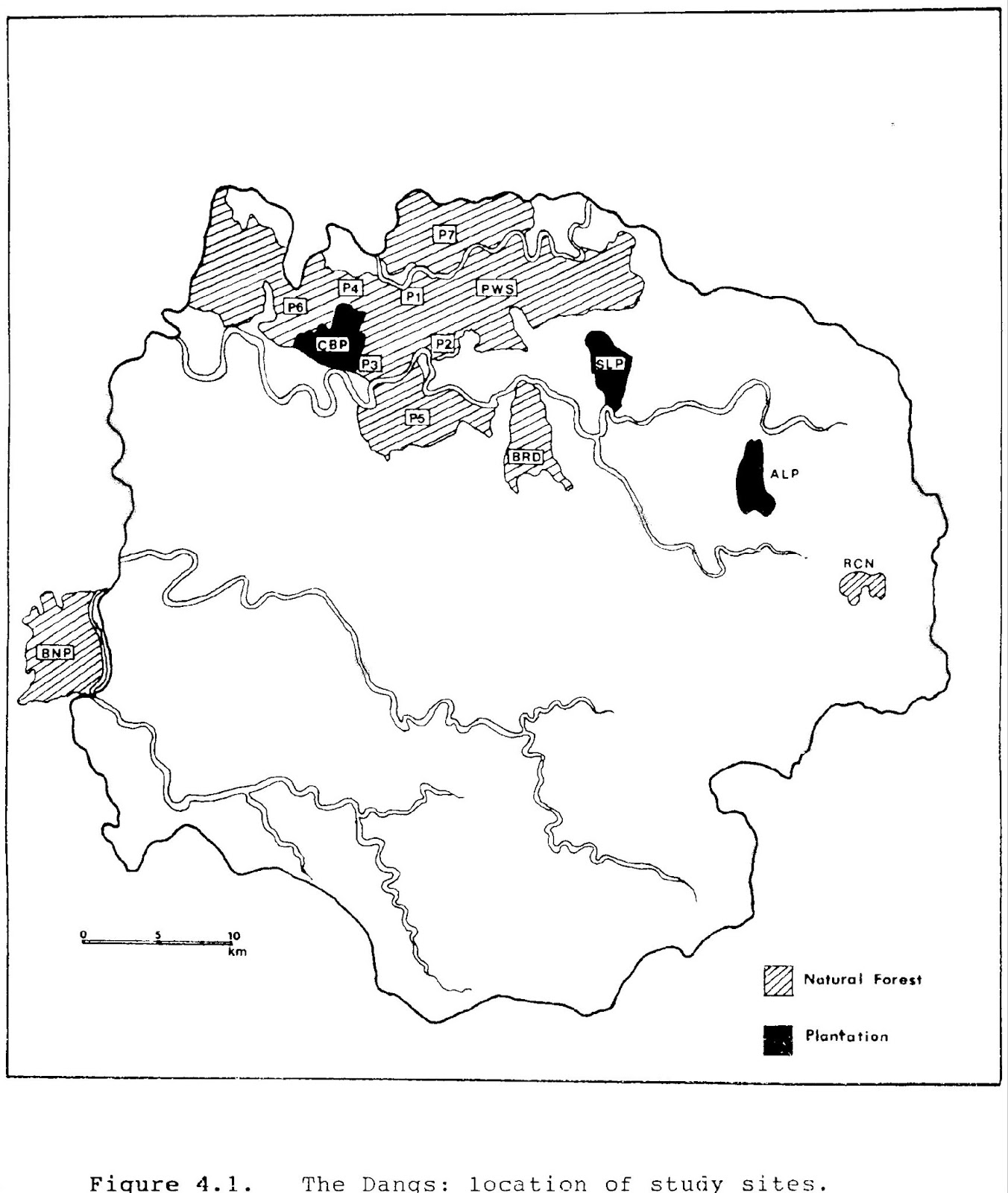
Figure S1: Sampled areas of Worah 1990 study area across Dang


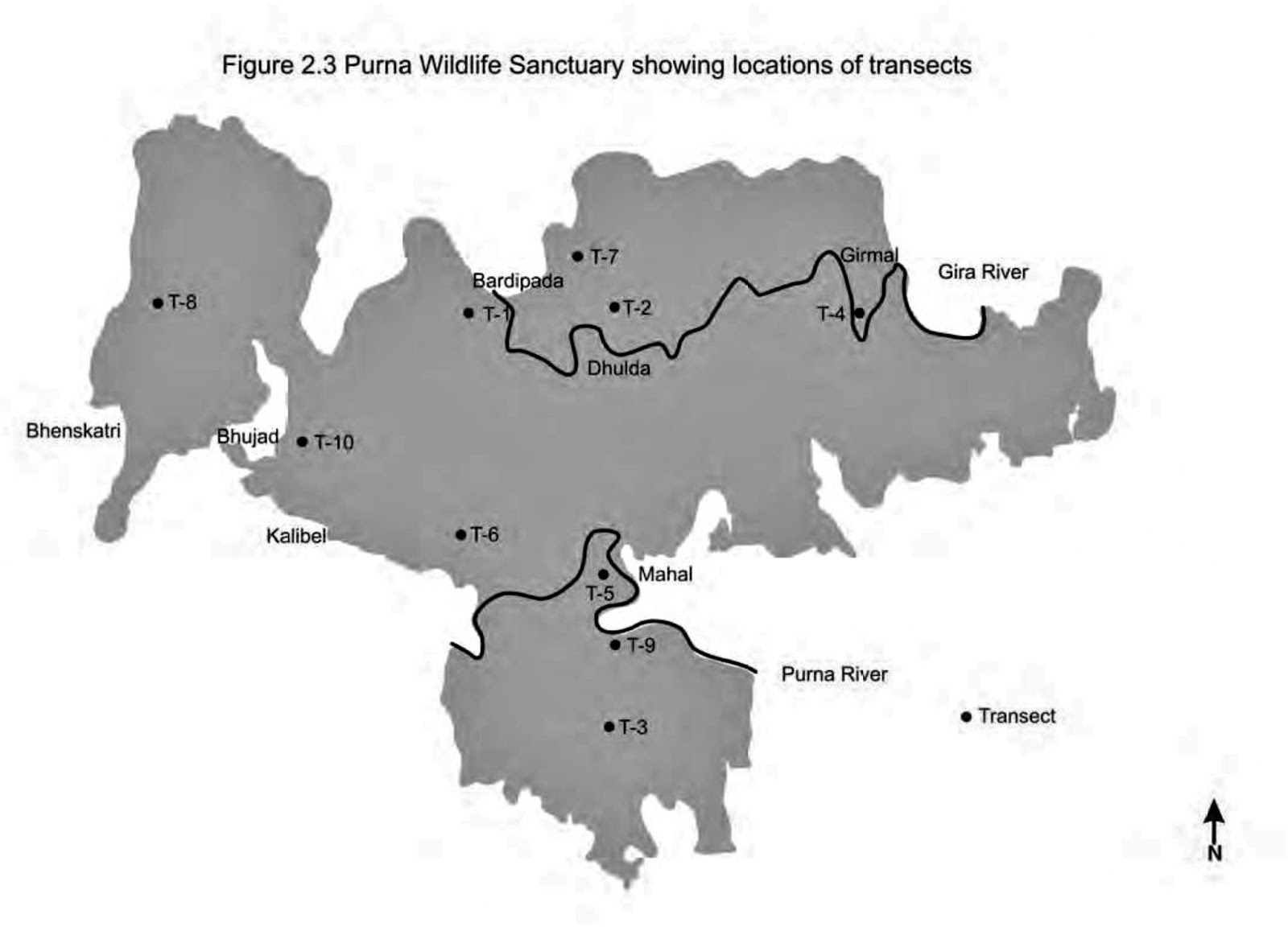


Figure S2: Locations of transects by Trivedi 2000 across Dang, including Purna Wildlife Sanctuary, north of Dang


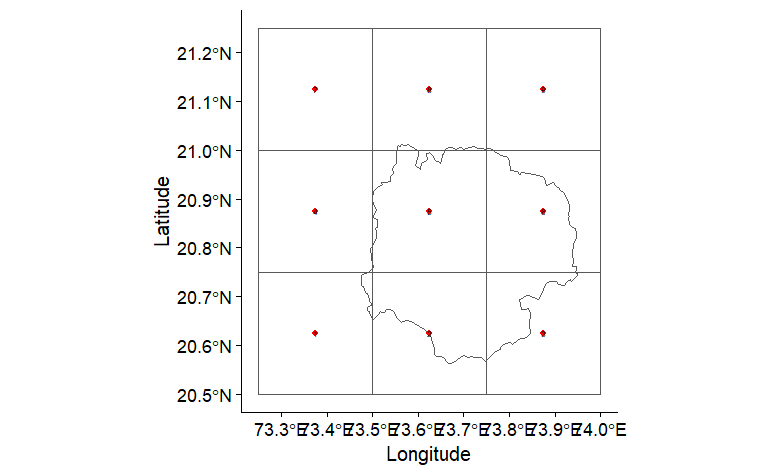


Figure S3: Locations/centroids of 0.25-degree grids around the Dang region (outline map) for which historic climate data from 1951 to 2019 based on Mishra *et al.* [*(2019)*](https://paperpile.com/c/VDaotW/UqNEz/?noauthor=1) was extracted for comparison with present data


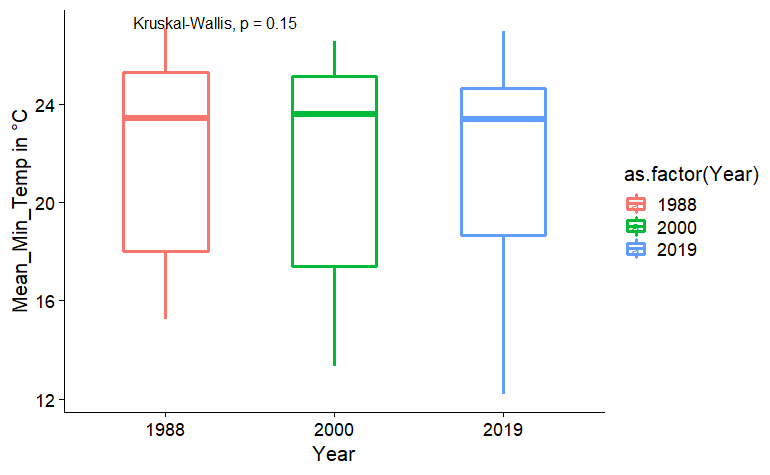


Figure S4a: Annual mean minimum temperature based on Mishra *et al.* [*(2019)*](https://paperpile.com/c/VDaotW/UqNEz/?noauthor=1) across Dang over three time periods closest to the survey years shows no significant change


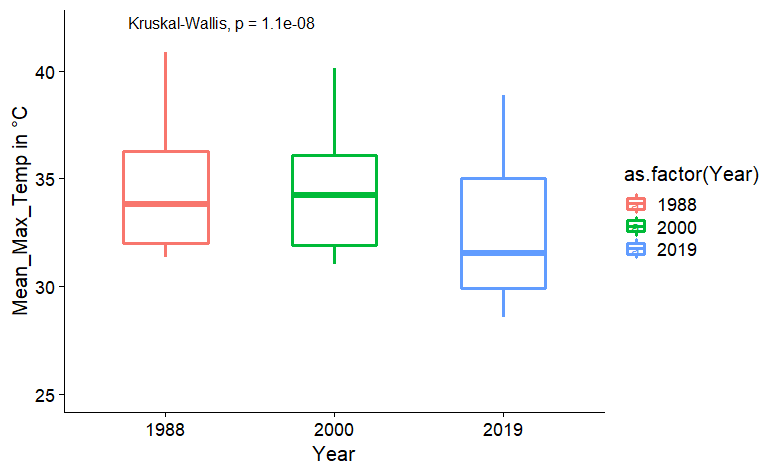


Figure S4b: Annual mean maximum temperature based on Mishra *et al.* [*(2019)*](https://paperpile.com/c/VDaotW/UqNEz/?noauthor=1) across Dang over three time periods closest to the survey years shows a significant change in 2019


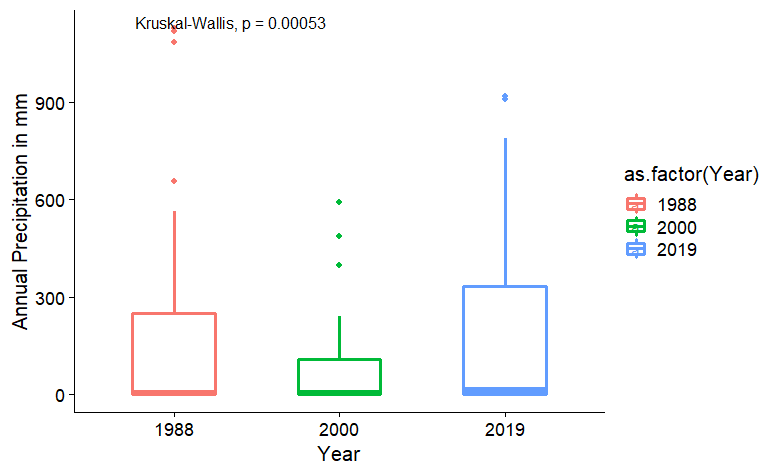


Figure S4c: Mean annual precipitation based on Mishra et al. [(2019)](https://paperpile.com/c/VDaotW/UqNEz/?noauthor=1)  across Dang over three time periods closest to the survey years shows no significant change


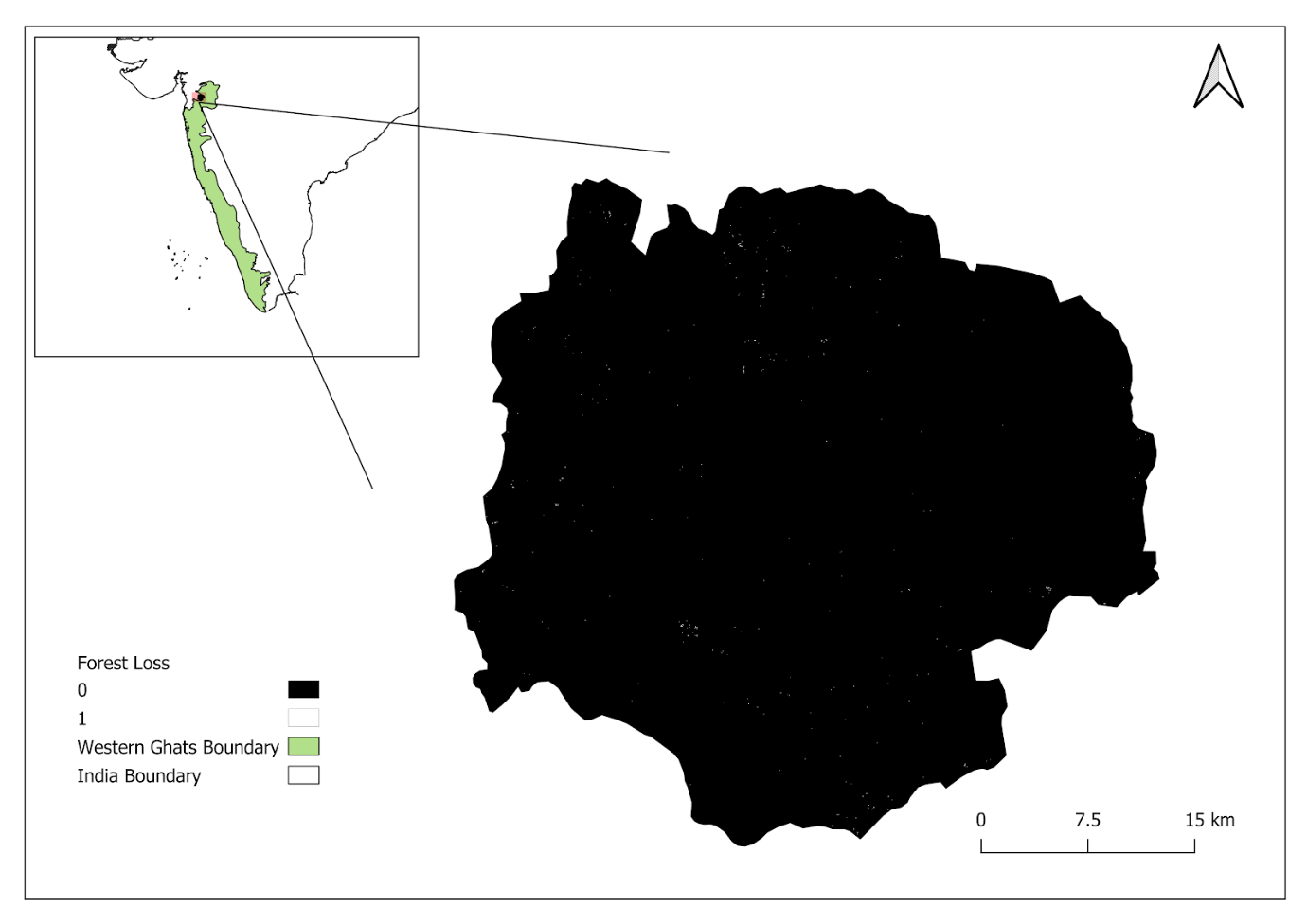


Figure S5: Forest loss across Dang from 2000 to 2019 appears minimal when assessed with global data products (Source: Hansen/UMD/Google/USGS/NASA). Here, pixel value 1 represents forest loss; 0 represents no perceivable change.


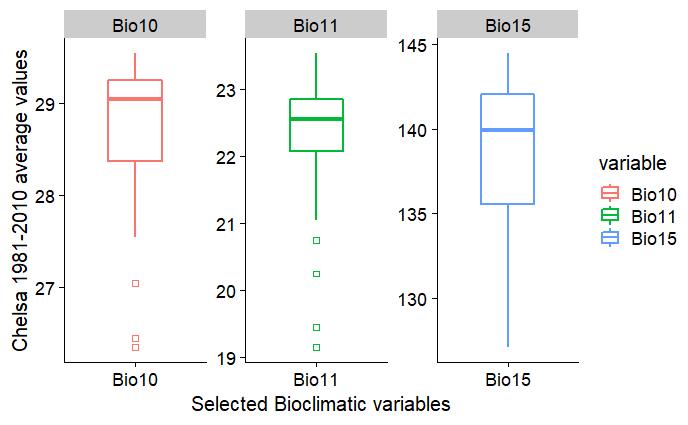
Figure S6: Selected Bioclimatic variables from 1981-2010 Chelsea for the Forest Owlet resurvey grids across Dangs (Bio10 - Mean temperature of the warmest quarter, Bio11 - Mean temperature of the coldest quarter, Bio15 - Precipitation Seasonality (Coefficient of Variation)


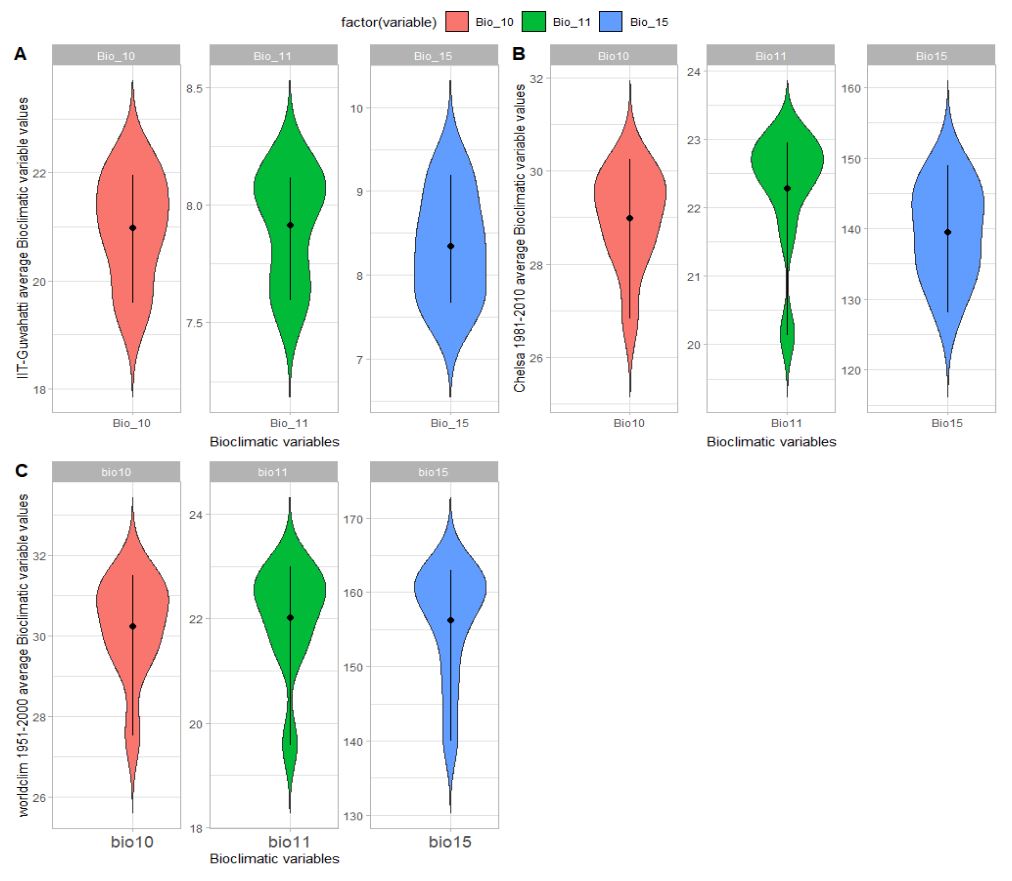


Figure S7:  Comparison of selected bio-climatic variable values based on Chelsea [(Karger et al., 2021)](https://paperpile.com/c/VDaotW/Avbwy) (average of years 1981 to 2010) and Worldclim Climate data version 1.3 [(*Wordclim Climate Data Version 1.3*, 2004)](https://paperpile.com/c/VDaotW/0NLCN/?noauthor=1)(average of years 1951 to 2000) across nine grids of climate locations from Mishra et al.  [(2019)](https://paperpile.com/c/VDaotW/UqNEz/?noauthor=1) climate data
